# Extracellular fluid excess linked to reduced choroidal vascularity index in patients with chronic kidney disease

**DOI:** 10.1038/s41598-024-63444-7

**Published:** 2024-06-04

**Authors:** Minjae Kang, Jongrok Oh, Min Kim, Suk Ho Byeon, Sung Soo Kim, Joo Youn Shin

**Affiliations:** 1 Department of Ophthalmology, Institute of Vision Research, Yongin Severance Hospital, Yonsei University College of Medicine, Yongin, Republic of Korea; 2 Department of Ophthalmology, Institute of Vision Research, Gangnam Severance Hospital, Yonsei University College of Medicine, Seoul, Republic of Korea; 3 Department of Ophthalmology, Institute of Vision Research, Severance Hospital, Yonsei University College of Medicine, Seoul, Republic of Korea

**Keywords:** Optics and photonics, Medical research

## Abstract

Extracellular fluid (ECF) excess is common in patients with chronic kidney disease (CKD). This study (involving 284 patients with CKD) explored the association between choroidal vascularity index (CVI) and ECF excess. We categorised patients into three groups based on extracellular water/total body water: normal, mildly overhydrated, and severely overhydrated. The more severe ECF status was associated with a lower CVI after adjustment (B = − 0.902, *p* = 0.001). In non-diabetic patients, both vascular luminal (LA, *p* < 0.001) and stromal areas (SA, *p* = 0.003) were significantly reduced in patients with severe ECF excess compared to others, whereas diabetic patients showed no significant differences in LA (*p* = 0.96) and SA (*p* = 0.86) based on ECF excess status. These findings suggest that ECF status may influence CVI in patients with CKD, underscoring the need for further research to clarify its direct impact on choroidal changes.

## Introduction

The choroid is a dense network of blood vessels and pigmented stroma between the retina and the sclera. It plays a crucial role in supplying oxygen and nutrients to the outer retina, light absorption, and thermoregulation^1^. The choroidal circulation (responsible for 85% of eye blood flow and is mainly controlled by sympathetic innervation) lacks autoregulation^2^. Although structural analysis of the choroidal vasculature has historically been challenging due to its inaccessibility, enhanced depth imaging (EDI)—optical coherence tomography (OCT) has enabled high-resolution visualisation of subtle vascular changes in choroidal structures^3^.

Choroidal Vascularity Index (CVI) was recently proposed as a novel marker, complementing subfoveal choroidal thickness (SFCT) in assessing choroidal changes using OCT^4^. It evaluates the choroidal vascular system by quantifying both its vascular (luminal) and stromal components. Notably, CVI measurements are a more stable parameter than SFCT, with reduced inter-assay variability and lower dependence on physiological factors^5^.

Chronic kidney disease (CKD), affecting over 10% of the global population, has emerged as one of the leading causes of mortality worldwide^6^. Some studies have investigated the impact of CKD on the choroid. Patients with CKD exhibit choroidal thinning compared to those with normal kidney function, and this thinning is correlated with the level of systemic inflammation and the degree of renal dysfunction^7,8^. A reduction in CVI has also been observed in patients with CKD, both in those with hypertension and diabetes mellitus (DM)^5,9^. These findings suggest that choroidal alterations observed in individuals with CKD might indicate broader systemic microvascular damage and potentially reflect underlying kidney injury.

Extracellular fluid (ECF) excess is common in individuals with CKD. ECF excess increases as the estimated glomerular filtration rate (eGFR) declines and has been identified as an independent factor influencing structural cardiac and vascular alterations in the course of CKD^10^. Chronic fluid overload has been observed even in the early stages of CKD, and this condition has been linked to both mortality and cardiovascular morbidity^11,12^. Some studies have suggested changes in SFCT and CVI before and after haemodialysis in patients with kidney failure^13^ who have ECF excess^14,15^. However, the associations between ECF excess and choroidal morphology have not been reported in an independent context. This study aimed to explore the association between the CVI and the state of ECF excess in individuals with CKD, along with related factors.

## Results

### Baseline characteristics

The average age was 61.72 years, and 58.8% were male. The mean extracellular water (ECW)/total body water (TBW) ratio was 0.391, and the ECF excess status was distributed as follows: 43.3% for normal, 31.7% for mildly overhydrated, and 25% for severely overhydrated status. The mean SFCT was 259.43 μm, and the mean CVI was 65.87%.

We analysed baseline characteristics by categorising the participants into three groups based on ECF excess status (Table 1). Concerning OCT parameters, the luminal area (LA) (*p* = 0.050) and stromal areas (SA) (*p* = 0.25) did not exhibit significant differences among the groups, whereas the CVI showed a significant difference (*p* < 0.001). The severely overhydrated status group had a significantly lower CVI compared to the normal (*p* < 0.001) and mildly overhydrated groups (*p* = 0.006). Furthermore, ECW/TBW exhibited a negative correlation with CVI, with a Pearson correlation coefficient of − 0.190 (*p* = 0.001).

**Table 1:** Baseline characteristics according to extracellular fluid excess status.

	ECF excess status			p-value
	Normal	Mild	Severe	
N, %	123 (43.3)	90 (31.7)	71 (25)	
Age, years	56.19 ± 14.59	65.51 ± 11.58^†^	66.51 ± 10.67^‡^	< 0.001*
Sex, male, n (%)	87 (70.7)	43 (47.8)	37 (52.1)	0.001*
Smoking status, n (%)				0.007*
Non	55 (44.7)	55 (61.1)	45 (63.4)	
Ex-smoker	46 (37.4)	30 (33.3)	22 (31.0)	
Smoker	22 (17.9)	5 (5.6)	4 (5.6)	
BMI, kg/m^2^	26.21 ± 4.08	24.96 ± 4.04	24.13 ± 3.84^‡^	0.002*
HTN (n, %)	112 (91.1)	77 (85.6)	58 (81.7)	0.16
DM (n, %)	71 (57.7)	61 (67.8)	40 (56.3)	0.23
SBP, mmHg	126.04 ± 19.21	130.73 ± 17.74	137.69 ± 21.86^‡^	< 0.001*
eGFR, mL/min/1.73 m^2^	52.55 ± 22.30	43.97 ± 22.61^†^	32.26 ± 21.63^‡§^	< 0.001*
LDL-cholesterol, mg/dL	92.91 ± 27.14	91.65 ± 31.04	99.00 ± 33.80	0.29
CRP, mg/dL	4.82 ± 13.79	1.85 ± 2.49	3.47 ± 8.92	0.13
CKD stage, n (%)				< 0.001*
1	0 (0)	0 (0)	6 (8.5)	
2	48 (39.0)	22 (24.4)	6 (8.5)	
3	49 (39.8)	31 (34.4)	17 (23.9)	
4	25 (20.3)	35 (38.9)	33 (46.5)	
5	1 (0.8)	2 (2.2)	9 (12.7)	
HbA1c, mmol/mol	6.37 ± 1.05	6.40 ± 0.96	6.38 ± 1.09	0.97
DR grade, n (%)				0.09
No DR	55 (77.5)	41 (67.2)	21 (52.5)	
NPDR	8 (11.3)	12 (19.7)	12 (30.0)	
PDR	8 (11.3)	8 (13.1)	7 (17.5)	
ECW/TBW	0.377 ± 0.018	0.392 ± 0.004^†^	0.414 ± 0.051^‡§^	< 0.001*
*OCT parameters*				
CVI, %	66.75 ± 3.11	65.85 ± 2.78	64.37 ± 3.06^‡§^	< 0.001*
Luminal area	177.93 ± 103.38	173.15 ± 106.83	141.59 ± 89.58	0.05
Stromal area	88.18 ± 49.67	89.01 ± 56.34	76.81 ± 46.67	0.25
SFCT, μm	277.03 ± 85.35	259.98 ± 87.91	228.24 ± 80.02^‡^	0.001*

ECF, extracellular fluid; BMI, body mass index; HTN, hypertension; DM, diabetes mellitus; SBP, systolic blood pressure; eGFR, estimated glomerular filtration rate; LDL, low-density lipoprotein; CRP, C-reactive protein; CKD, chronic kidney disease; HbA1c, glycated haemoglobin; DR, diabetic retinopathy; NPDR, non-proliferative diabetic retinopathy; PDR, proliferative diabetic retinopathy; ECW/TBW, extracellular water to total body water ratio; OCT, optical coherence tomography; CVI, choroidal vascularity index; SFCT, subfoveal choroidal thickness.

*p < 0.05 in ANOVA, †*p* < 0.05 between normal and mild group, ‡*p* < 0.05 between normal and severe group, §*p* < 0.05 between mild and severe group.

### Association of CVI with ECF excess status

In the univariate linear regression analysis, the presence of DM (*p* = 0.002), systolic blood pressure (SBP) (*p* = 0.003), eGFR (*p* < 0.001), and the ECF excess status (*p* < 0.001) were associated with CVI. In the multivariate analysis, after adjusting for age (*p* = 0.99), sex (*p* = 0.09), presence of DM (B = − 1.149, *p* = 0.011), SBP (*p* = 0.34), eGFR (B = 0.036, *p* < 0.001), low-density lipoprotein (LDL)-cholesterol (*p* = 0.76) and Haemoglobin A1c (HbA1c) (*p* = 0.96), a significant association between more severely overhydrated status and lower CVI was observed (B = − 0.902, *p* = 0.001) (Table 2).

**Table 2:** Linear regression analysis of variables with choroidal vascularity index.

	Univariate			Multivariate		
	B	95% CI	*p *value	B	95% CI	*p *value
Age	− 0.020	− 0.047 to 0.007	0.14	0.000	− 0.032 to 0.031	0.99
Sex	0.155	− 0.589 to 0.899	0.68	0.652	− 0.099 to 1.402	0.09
Smoking status	− 0.055	− 0.593 to 0.482	0.84			
BMI	− 0.021	− 0.112 to 0.069	0.65			
HTN	0.066	− 1.022 to 1.154	0.91			
DM	− 1.160	− 1.897 to − 0.423	0.002*	− 1.149	− 2.027 to − 0.271	0.011*
SBP	− 0.009	− 0.046 to − 0.010	0.003*	− 0.009	− 0.028 to 0.009	0.34
eGFR	0.042	0.027 to 0.057	< 0.001*	0.036	0.018 to 0.053	< 0.001*
LDL-cholesterol	− 0.004	− 0.017 to 0.009	0.54	− 0.002	− 0.014 to 0.010	0.76
HbA1c	− 0.163	− 0.524 to 0.197	0.37	0.009	− 0.391 to 0.410	0.96
ECF excess	− 1.160	− 1.594 to − 0.727	< 0.001*	− 0.902	− 1.418 to − 0.387	0.001*

B, unstandardised coefficient; CI, confidence interval; BMI, body mass index; HTN, hypertension; DM, diabetes mellitus; SBP, systolic blood pressure; eGFR, estimated glomerular filtration rate; LDL, low-density lipoprotein; HbA1c, glycated haemoglobin; ECF, extracellular fluid.

*p < 0.05.

### Subgroup analysis: non-DM vs. DM patients

Since the regression analysis indicated that the presence of DM influenced the association between ECF excess status and CVI, the subjects were divided into two groups: a non-DM group and a DM group for further analysis. Of the 284 subjects, 112 were in the non-DM group, and 172 were in the DM group. Patients in the DM group were older (*p* < 0.001) and had a higher prevalence of hypertension (*p* = 0.029) than those in the non-DM group. In addition, body mass index (BMI) (*p* = 0.001), SBP (*p* = 0.002), CKD stage (*p* < 0.001), and HbA1c levels (*p* < 0.001) were higher in the DM group. LDL-cholesterol (*p* = 0.010) and CVI (*p* = 0.004) were lower in the DM group than in the non-DM group (Supplemental Table 1).

In the non-DM and DM groups, severe excess of ECF was significantly associated with lower CVI (Table 3). In the non-DM group, even after adjusting for age, sex, SBP, eGFR and LDL-cholesterol, there remained an association of ECF excess status with lower CVI (B = − 1.500, *p* = 0.007). In the DM group, after further adjustment for HbA1c level and diabetic retinopathy (DR) grade, this association remained significant (B = − 0.658, *p* = 0.025).

**Table 3:** Association between choroidal vascularity index and extracellular fluid excess status in the non-diabetic group and the diabetic group.

	Non-DM			DM		
	B	95% CI	p-value	B	95% CI	p-value
Age	− 0.007	− 0.066 to 0.051	0.80	0.025	− 0.013 to 0.062	0.19
Sex	0.509	− 0.886 to 1.904	0.47	0.352	− 0.511 to 1.214	0.42
SBP	− 0.015	− 0.054 to 0.023	0.42	− 0.007	− 0.028 to 0.014	0.52
eGFR	0.007	− 0.031 to 0.044	0.73	0.037	0.018 to 0.057	< 0.001*
LDL-cholesterol	− 0.007	− 0.030 to 0.015	0.51	0.000	− 0.015 to 0.014	0.98
HbA1c				0.041	− 0.414 to 0.496	0.86
DR grade				0.077	− 0.519 to 0.673	0.80
ECF excess status	− 1.500	− 2.588 to − 0.412	0.007*	− 0.658	− 1.231 to − 0.084	0.025*

DM, diabetes mellitus; B, unstandardised coefficient; CI, confidence interval; SBP, systolic blood pressure; eGFR, estimated glomerular filtration rate; LDL, low-density lipoprotein; HbA1c, glycated haemoglobin; DR, diabetic retinopathy; ECF, extracellular fluid.

*p < 0.05.

We analysed how the components contributing to CVI, specifically the LA and SA, differed according to the ECF excess status (Table 4). In the non-DM group, the severely overhydrated group had significantly lower values for both the LA and SA compared to the normal (*p* < 0.001 for LA, *p* = 0.005 for SA) and mildly overhydrated groups (*p* = 0.002 for LA, *p* = 0.013 for SA). In contrast, in the DM group, there were no significant differences in the LA (*p* = 0.96) and SA (*p* = 0.86) based on the ECF excess status.

**Table 4:** Comparison of optical coherence tomography parameters based on extracellular fluid excess status in the non-diabetic and diabetic groups.

	ECF excess status			p-value
	Normal	Mild	Severe	
*Non-DM group*				
CVI	67.72 ± 3.51	67.24 ± 2.37	64.02 ± 3.28^‡§^	< 0.001*
LA	195.26 ± 107.76	191.93 ± 100.31	105.23 ± 68.49^‡§^	< 0.001*
SA	92.08 ± 47.97	93.31 ± 52.70	58.41 ± 35.97^‡§^	0.003*
*DM group*				
CVI	66.04 ± 2.57	65.18 ± 2.73	64.65 ± 2.89^‡^	0.027*
LA	165.24 ± 98.90	164.22 ± 109.46	169.76 ± 101.24	0.96
SA	85.33 ± 51.04	86.97 ± 58.29	91.07 ± 49.36	0.86

ECF, extracellular fluid; DM, diabetes mellitus; CVI, choroidal vascularity index; LA, luminal area; SA, stromal area.

*p < 0.05 in ANOVA, †*p* < 0.05 between normal and mild group, ‡*p* < 0.05 between normal and severe group, §*p* < 0.05 between mild and severe group.

## Discussion

The current study demonstrated a significant association between ECF excess and reduced CVI in patients with CKD. This finding was consistent across both the non-DM and DM groups. In the non-DM group, individuals with severe ECF excess exhibited notably lower LA and SA than those with normal or mild ECF excess; however, in DM patients, there was no significant difference in LA and SA based on ECF excess status.

Several studies have explored the effect of renal function on the choroid in patients with CKD. Thinner choroids have been observed in patients with CKD compared to those with normal renal function^7,8^, and a reduction in CVI has also been documented in patients with CKD^5,9^. Chorioretinal thinning in CKD is associated with a lower eGFR and increased circulating levels of inflammatory markers, such as C-reactive protein, interleukin-6, asymmetric dimethylarginine, and endothelin-1. These findings suggest that choroidal changes in patients with CKD are correlated with systemic inflammation, endothelial dysfunction, and vasoconstriction^16^. In the present study, multivariate regression analysis revealed an association between reduced renal function (lower eGFR) and decreased CVI, which is consistent with previous findings.

ECF excess is prevalent in patients with CKD, increasing as eGFR declines^10,11^. This chronic fluid overload is evident even in the early CKD stages and has been identified as a significant independent factor contributing to structural cardiac and vascular changes throughout the disease course^10^. The clinical assessment of the ECF status is relatively difficult because the physical signs of oedema are of limited value in the diagnosis of ECF excess^17^. In this study, we used bioelectrical impedance analysis (BIA) to assess the ECF status by measuring the impedance of the body to applied electric currents of different frequencies^18^, and showed that ECF excess is independently linked to decreased CVI in patients with CKD after adjusting for multiple confounding factors.

The association between ECF excess and lower CVI can be attributed to several potential mechanisms. Diminished renal function leads to reduced urinary excretion of sodium and fluid when ECF excess is present, prompting the activation of the intrarenal renin–angiotensin–aldosterone system, which exacerbates ECF excess. Angiotensin II acts as a powerful vasoconstrictor and promotes sympathetic overactivity through the regulation of central sympathetic outflow^19,20^. This interaction could potentially contribute to the decrease in the CVI. Furthermore, the persistent ECF excess results in pathologic mechanical stimuli on vascular endothelial and smooth muscle cells^11^. ECF overload is significantly linked to microinflammation and markers of endothelial dysfunction^21^, contributing to increased superoxide production and reduced nitric oxide (NO) bioavailability in patients with CKD^22^. Accelerated atherosclerosis, vasoconstriction, endothelial dysfunction, and proliferation of smooth muscle cells in vessel walls potentially lead to choroidal changes in the context of ECF excess. Further research is warranted to comprehensively understand the mechanisms underlying the relationship between ECF excess and CVI in patients with CKD.

ECF excess status was associated with a decrease in CVI in the DM and non-DM groups. However, when the SA and LA were analysed separately, the DM and non-DM groups exhibited different patterns. In the non-DM group, individuals with severe ECF excess exhibited significantly reduced LA and SA compared to those with normal or mild ECF excess. There was a more prominent decrease in the LA, which might have led to a secondary reduction in the SA, potentially leading to a decrease in the CVI. In DM patients, no significant differentiation was observed in the LA and SA based on ECF excess status. Despite the lack of statistical significance, patients with severe ECF excess in the DM group exhibited larger LA and SA than those with a normal ECF status or mild excess. The significant decrease in CVI within the ECF excess group in the DM group may be explained by the relatively modest increase in the LA compared to the greater increase in the SA. Given the differences in baseline characteristics between the DM and non-DM groups, it is challenging to interpret the results as a direct comparison; however, these findings suggest the possibility of distinct choroidal changes in response to ECF excess status between the non-DM and DM groups.

This study had some limitations. First, the causative relationship could not be determined due to the cross-sectional study design. However, longitudinal studies are required to confirm these conclusions. Second, although patients taking diuretics or steroids were excluded from the analysis, the influence of other medications (for example, angiotensin-converting enzyme inhibitors, beta-blockers, and statins) could potentially affect the results. Third, information regarding the ocular factors that could affect the choroid, such as intraocular pressure or refractive error, was not available. However, CVI is less affected by ocular factors than SFCT^4,5^. Despite these limitations, we demonstrated that the non-traditional factor of ECF excess independently affects the choroid in patients with CKD. Larger studies focusing on specific patient groups are necessary to better understand the longitudinal changes during the progression of CKD. In addition, considering the known association between ECF excess and cardiovascular morbidity and mortality^11,12^, exploring whether CVI could serve as a biomarker reflecting systemic vascular status would be an interesting avenue of research.

In summary, this study demonstrated a significant and independent association between ECF status, influenced by renal function, and the CVI in patients with CKD. These findings imply that ECF status could play a role in influencing CVI in patients with CKD, prompting the need for further research to elucidate the direct impact of ECF status on choroidal changes.

## Methods

The study protocol was approved by the Institutional Review Board of the Yonsei University Health System Clinical Trial Centre. The study was conducted according to the principles of the Declaration of Helsinki. All the patients provided written informed consent before participating in the study (IRB No. 4–2013-0661). All the methods were carried out in accordance with relevant guidelines and regulations.

### Study populations

The participants in this study were selected from the Cardiovascular and Metabolic Diseases Etiology Research Center High-Risk Cohort (CMERC-HI, https://clinicaltrials.gov/NCT02003781) study. This cohort consisted of individuals at a high risk of developing cardiovascular disease (CVD) who did not exhibit symptomatic CVD at baseline, and the participants were enrolled from 2013 to 2018. The study protocol will be reported in detail elsewhere^23^. Subjects were deemed to have cardiovascular risk factors and were enrolled in the CMERC-HI cohort study if they had any of the following: high-risk hypertension (eGFR > 60 mL/min/1.73 m2 with damage to at least one target organ or an eGFR ≤ 60 mL/min/1.73 m2); DM with albuminuria; anuric kidney failure requiring dialysis; a relative who sustained an acute myocardial infarction when aged younger than 55 years (if male) or 65 years (if female); asymptomatic atherosclerotic disease (abdominal aorta diameter ≥ 3 cm or ankle-brachial index > 0.9, carotid plaque or carotid intima-media thickness ≥ 0.9 mm, an asymptomatic old cerebrovascular accident, or > 30% stenosis in at least one major coronary artery); rheumatoid arthritis, age > 40 years, and taking methotrexate and steroids; atrial fibrillation with a CHA2DS-VASc score ≥ 1; and a history of kidney transplantation more than 3 months beforehand. Subjects aged > 20 years who met at least one inclusion criterion were enrolled in the study. Subjects with any of the following were excluded: acute myocardial infarction, acute coronary syndrome, or symptomatic coronary artery disease, or a history of either of these diseases; symptomatic peripheral artery disease or heart failure, or a history of either of these diseases; expected survival of less than 6 months for a non-cardiovascular reason (e.g., cancer or sepsis); history of contrast allergy or side effects related to contrast materials; and current pregnancy or lactation.

Participants who were enrolled in this cohort between 2013 and 2016 had undergone ophthalmic examination at the time of enrolment. Among these, OCT imaging using EDI mode was conducted in 2015, and the images of corresponding subjects were analysed (n = 372). We excluded participants who (1) had medications (diuretics or steroids) or ophthalmic conditions (other than DR) that could potentially impact the choroid, such as central serous chorioretinopathy, age-related macular degeneration, retinal vessel occlusion, glaucoma, chorioretinal scarring, or myopic macular degeneration (n = 28); (2) received laser photocoagulation or anti-vascular endothelial growth factor treatment for DR (n = 29); (3) had undergone cataract surgery or vitrectomy within 3 months (n = 1); (4) exhibited significant media opacity or poor image quality (n = 3); (5) were on maintenance dialysis or had undergone kidney transplantation (n = 22); (6) had inadequately measured or missing laboratory or BIA measurements (n = 5). In total, we enrolled 284 patients in this study.

### Clinical, laboratory data collection

We collected clinical and laboratory data at the time of enrolment. Participants underwent a standardised interview that covered inquiries about established diagnoses of hypertension, DM, and CKD, ongoing treatments for these conditions, and smoking history. Anthropometric measurements, including body height and weight, were recorded. HbA1c, blood glucose, lipid (LDL-cholesterol), blood urea nitrogen, and creatinine levels were measured in blood samples collected after overnight fasting. CKD stages were determined based on the eGFR. SBP and diastolic blood pressure were measured.

### Ophthalmic data collection

Fundus photography (Visucam NM/FA; Carl Zeiss Meditec, Jena, Germany) and spectral domain OCT (Spectralis HRA OCT; Heidelberg Engineering GmbH, Dossenheim, Germany) were performed on the same day. The images were assessed to identify ophthalmic conditions and evaluate image quality. DR was graded as no DR, non-proliferative DR (NPDR), or proliferative DR (PDR).

### Measurement of ECF status

ECF excess status was determined using a BIA (InBody 720 Body Composition Analyzer; Biospace, Seoul, Korea) at the time of CMERC-HI enrolment. This body composition analyser employs a 4-pole, 8-point tactile electrode system, performing 30 impedance measurements at six frequencies. TBW, ECW, and intracellular water were calculated.

ECF excess was determined by calculating the ratio of ECW to TBW, denoted as ECW/TBW. Based on this ratio, ECF excess status was categorised into three groups: normal (ECW/TBW < 0.390), mildly overhydrated (0.390 ≤ ECW/TBW < 0.400), and severely overhydrated status (ECW/TBW ≥ 0.400)^11^.

### Measurement of CVI

**Figure 1:**
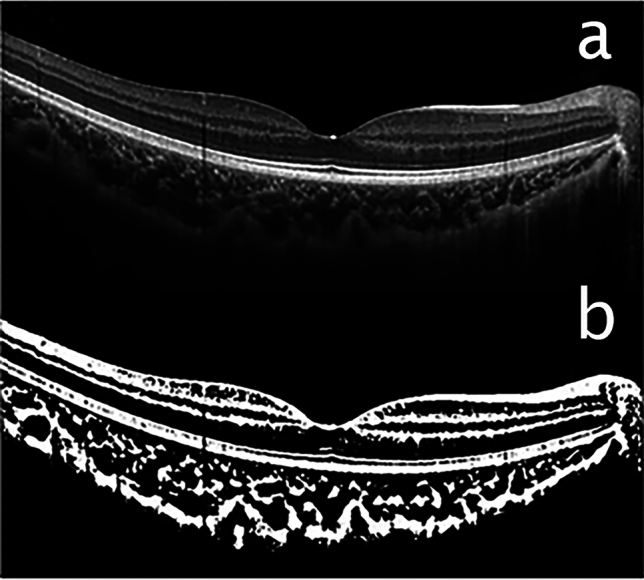
Representative images of processing to calculate the choroidal vascularity index (CVI) using enhanced depth image (EDI)– optical coherence tomography (OCT) scans. (**a**) original EDI-OCT image. (**b**) binarization of EDI-OCT image for calculation of CVI.

The CVI was measured using established methods with minor modifications^4,24^. EDI—OCT images that traversed the fovea were selected for analysis. Image binarization and segmentation were performed using the ImageJ software, version 1.47 (Bethesda, MD, USA; http://imagej.nih.gov/ij/). The total choroidal area (TCA) was determined as the chosen region within the subfoveal choroid, spanning a width of 1500 μm (with 750 μm extending both nasally and temporally from the fovea). The vascular LA was defined as the area occupied by dark pixels, whereas the SA was defined as the area occupied by light pixels. The CVI was defined as the ratio of LA to TCA (Fig. 1).

### Statistical analysis

Baseline characteristics and OCT parameters were compared based on the ECF excess status using a One-way Analysis of Variance with Bonferroni's method for continuous variables and the chi-squared test for categorical variables. For comparisons between the non-DM and DM groups, independent t-test and chi-squared test were used. Pearson correlation analysis was used to evaluate the relationship between ECW/TBW and CVI. Multivariate linear regression analysis was conducted to determine the association between ECF excess status and CVI. Parameters that showed significant associations with the CVI in the univariate analysis and were considered clinically relevant were chosen as independent variables. Additionally, given the significant correlation found between the ECW/TBW, age was included as an adjusted variable in the analysis^25^. Statistical analysis was performed using the Statistical package for the social sciences software (SPSS statistics 22 for Windows; SPSS, Inc., IBM, Somers, NY, USA). Statistical significance was set at *p* < 0.05.

### Supplementary Information

Supplementary Information.

## Data Availability

The datasets generated during and/or analysed during the current study are available from the corresponding author on reasonable request.
