# Supplementary material for: Extracellular fluid excess linked to reduced choroidal vascularity index in patients with chronic kidney disease
Source: Sci Rep. 2024 Jun 4;14:12769. doi: 10.1038/s41598-024-63444-7 (PMC11150457; doi:10.1038/s41598-024-63444-7)
Supplement: Supplementary file 1 — Supplementary Information. [file 41598_2024_63444_MOESM1_ESM.docx]

**Extracellular Fluid Excess Linked to Reduced Choroidal Vascularity Index in Patients with Chronic Kidney Disease**

Minjae Kang, Jongrok Oh, Min Kim, Suk Ho Byeon, Sung Soo Kim, Joo Youn Shin

Supplement table 1. Baseline characteristics between the non-diabetic and diabetic groups

|  | Total | Non-DM | DM | P-value |
| --- | --- | --- | --- | --- |
| n | 284 | 112 | 172 | NA |
| Age, years | 61.72 ± 13.63 | 55.93 ± 14.25 | 65.49 ± 11.80 | <0.001* |
| Sex, male (n, %) | 167(58.8) | 64 (57.1) | 103 (59.9) | 0.71 |
| Smoking status  Non  Ex-smoker  Smoker | 155 (54.6)  98 (34.5)  31 (10.9) | 67 (59.8)  37 (33.0)  8 (7.1) | 88 (51.2)  61 (35.5)  23 (13.4) | 0.18 |
| BMI, kg/m^2^ | 25.29 ± 4.09 | 24.30 ± 4.06 | 25.92 ± 3.99 | 0.001* |
| HTN (n, %) | 247 (87.0) | 91 (81.3) | 156 (90.7) | 0.029* |
| DM (n, %) | 172 (60.6) | 0 | 172 | NA |
| SBP, mmHg | 130.44 ± 19.95 | 125.88 ± 19.53 | 133.41 ± 19.70 | 0.002* |
| eGFR, mL/min/1.73 m^2^ | 44.98 ± 23.57 | 45.35 ± 22.73 | 44.74 ± 24.18 | 0.83 |
| LDL-cholesterol, mg/dL | 94.01 ± 30.15 | 100.17 ± 31.10 | 90.23 ± 29.17 | 0.010* |
| CRP, mg/dL | 3.50 ± 10.13 | 4.31 ± 11.69 | 2.98 ± 9.01 | 0.306 |
| CKD stage  1  2  3  4  5 | 6 (2.1)  76 (26.8)  97 (34.2)  93 (32.7)  12 (4.2) | 0 (0)  28 (25)  54 (48.2)  20 (17.9)  10 (8.9) | 6 (3.5)  48 (27.9)  43 (25.0)  73 (42.4)  2 (1.2) | <0.001* |
| HbA1c, mmol/mol | 6.38 ± 1.03 | 5.75 ± 0.80 | 6.79 ± 0.95 | <0.001* |
| DR grade  No DR  NPDR  PDR | 117 (68.0)  32 (18.6)  23 (13.4) | NA | 117 (68.0)  32 (18.6)  23 (13.4) | NA |
| ECW/TBW | 0.391 ± 0.032 | 0.387 ± 0.035 | 0.393 ± 0.029 | 0.16 |
| ECF excess status  Normal  Mild  Severe | 123 (43.3%)  90 (31.7%)  71 (25.0) | 52 (46.4)  29 (25.9)  31 (27.7) | 71 (41.3)  61 (35.5)  40 (23.3) | 0.23 |
| CVI, % | 65.87 ± 3.13 | 66.57 ± 3.54 | 65.41 ± 2.75 | 0.004* |
| SFCT, μm | 259.43 ± 86.79 | 267.42 ± 88.20 | 254.19 ± 85.72 | 0.21 |

DM, diabetes mellitus; NA, not applicable; HTN, hypertension; BMI, body mass index; SBP, systolic blood pressure; ECW/TBW, extracellular water to total body water ratio; ECF, extracellular fluid; eGFR, estimated glomerular filtration rate; LDL, low-density lipoprotein; CRP, C-reactive protein; CKD, chronic kidney disease; DR, diabetic retinopathy; NPDR, non-proliferative diabetic retinopathy; PDR, proliferative diabetic retinopathy; HbA1c, glycated haemoglobin; CVI, choroidal vascularity index; SFCT, subfoveal choroidal thickness.

*p<0.05
